# The Equity Imperative: Transforming Research Coproduction for Impact: Comment on "Research Coproduction: An Underused Pathway to Impact"

**DOI:** 10.34172/ijhpm.8875

**Published:** 2025-01-21

**Authors:** Kathleen P. Conte, Marisa Zapata

**Affiliations:** ^1^OHSU-PSU School of Public Health, Portland State University, Portland, OR, USA.; ^2^Homelessness Research and Action Collaborative, Portland State University, Portland, OR, USA.; ^3^Toulan School of Urban Studies and Planning, Portland State University, Portland, OR, USA.

**Keywords:** Coproduction, Equity, Futures Thinking, Collaboration, Participatory Research

## Abstract

Coproduction is not a new idea in implementation research, health research, or research in general. There is substantial scholarship that establishes its importance and provides guidance and examples for adoption. Given this, why do editorials like Rycroft-Malone and colleagues’ recent paper, "Research Coproduction: An Underused Pathway to Impact," continue to be published, and also, necessary? In this commentary, we discuss the importance of equity—not equality—as the underlying paradigm of coproduction research. We argue that it is the incomplete understanding and adoption of this equity paradigm by researchers and their institutions that inhibit coproduction from being fully realized and thus, impactful. We offer examples of what such a paradigm shift might look like, including futures thinking that yields difficult questions that must be addressed to dismantle systemic barriers to power redistribution.

*The idea of citizen participation is a little like eating spinach: no one is against it in principle because it is good for you… The applause is reduced to polite handclaps, however, when this principle is advocated by the have-not [minority individuals and communities]*^[1]^.* And when the have-nots define participation as re-distribution of power, the American consensus on the fundamental principle explodes into many shades of outright racial, ethnic, ideological, and political opposition. *

**Sherry Arnstein** ^1^

 Coproduction—ie, involving end-users in the process of research production—is a well-established concept in health research,^3^ and research more broadly.^4-6^ Rycroft-Malone and colleagues’^7^ editorial joins this literature by discussing coproduction as a necessary approach for improving research impact. They reiterate the idea that researchers should learn with and from non-research partners who are the intended beneficiaries of research to improve translation. Given the history of coproduction scholarship, it is pertinent to ask why editorials like Rycroft-Malone and colleagues’ continue to be necessary. In this commentary, we argue that answer lies in the ongoing struggle to fully embrace the equity principles that underpin coproduction. Our aim is to deepen appreciation of the significance of the paradigm shift that is required, from equality-driven coproduction to equity-driven coproduction, from research institutions and researchers.

## Equity not Equality

 Sherry Arnstein’s article, written over 55 years ago, marks a significant point in the birth of coproduction philosophies.^1^ Arnstein’s work laid out a vision for coproduction that was explicit that coproduction was about the redistribution of power. Hers and others’^3,8^ pioneering work in coproduction is fundamentally about *equity *and not *equality*. Equity means people get what they need based on their individual circumstances. Equality means uniformly distributing resources regardless of context. A co-productive model that is equal, per Rycroft-Malone et al, means “that all have an equal voice and role to play throughout the research lifecycle, including implementation.” An equity approach, however, recognizes that power and resources must be actively, purposefully and continuously redistributed throughout the research lifecycle, and that often, voices of those from marginalized groups must hold more weight. We contend that, to date, coproduction and its ability to facilitate research impact is under-realized because of a misalignment between this fundamental philosophy of coproduction as based in equity, and the goals of research institutions and of researchers themselves.

 Consider the rise in research institutions (ie, funders, universities, and think tanks) that now require coproduction or other forms of collaborative research. For governments, policy-makers, health care administrators, big business and others who already operate with substantial forms of their own power, coproduction with one another can lead to long-term, productive partnerships and knowledge sharing. Because their starting point is one steeped in power, there is no threat of disruption of the status quo. Agreeing that co-production is what people want to do is much easier when equality is the goal.

 Yet, as Arnstein described, the conversation and commitment changes when the coproduction goal is to share or cede power to marginalized communities^[2]^. And, marginalized communities are far more interested in equity than equality. It is not that researchers and their institutions are likely to explode into *outright “*racial, ethnic, ideological, and political opposition” to coproduction, however, opposition to power sharing and redistribution is baked into the operation of research systems, and therefore, much more covert and intractable. An equity-centered approach to research, coproduced or otherwise, involves disrupting the status quo. It involves identifying how power is held and maintained by institutions and the people who comprise them, and finding ways to redistribute that power to the people with whom we are doing coproduction. Next, we consider several examples of types of power redistribution, acknowledging this is an incomplete list but seeking to demonstrate what kinds of changes an equity lens requires.

## Examples of Power Redistribution

*Redistributing materials:* In coproduction spaces, there is substantial talk about transferring material resources to people during a research process. For example, hiring and training community members is an important strategy for integrating experiential expertise into research projects. But institutional processes can make hiring and reimbursements inordinately difficult. Researchers doing coproduction find they must become administratively adroit to accomplish tasks that outside an institutional context might be simple, such as purchasing and distributing gift cards or cash incentives. “Workarounds” and rule bending are common practices, though are rarely discussed as practical—and certainly not legitimate—strategies to overcoming administrative barriers.

 Changing resource distribution models within institutions is *hard* – it drains time and resources away from other power-shifting activities, eg, like thinking differently about who we engage as coproduction partners. For example, while hiring community members might promote equality, material redistribution would do more than provide compensation. Grants rarely provide the kinds of resources required—including time, money, and other materials resources—to meaningfully support community development of skills, infrastructure, and relationships that would facilitate *equitable *engagement with researchers. There are very few examples of full-time positions to support community members’ long-term participation in research. Instead payments are limited to hourly rates or short-term contracts that do not provide the kind of security and benefits that researchers fully employed on projects are likely to enjoy^[3]^.

 Material redistribution risks becoming a red herring for deep, meaningful changes in other ways that power is held and maintained. Because administrative reform is so challenging, it is easy to become distracted by these tasks without getting to the heart of power reform. These difficulties demonstrate how covert and intractable resistance to power change is: in the face of great administrative difficulties, projects are forced to act in ways where alignment is with the goals of the institution and not with the goals of coproduction. For example, coproduction at public universities involving partnerships with marginalized groups engaged in illegal activity, eg, undocumented immigrants, or individuals for whom research payments might negatively affect their ability to obtain/keep public benefits, may be all but impossible due to employment and taxation laws. This is despite the fact that engaging with such disenfranchised groups might yield useful solutions to address some of society’s critical issues, eg, in health, homelessness, and mental illness.

*Redistributing knowledge:* Notably, equal knowledge sharing throughout an entire research project is a problem inadequately addressed in the coproduction literature. Examples of partner involvement are notably missing from data analysis processes^9^ on the assumption that analysis requires specialized skills that only researchers hold.^10^ But if partners can be trained to collect data, they can be trained to analyze it as well. For that matter, researchers might be trained to understand how community partners make new knowledge. At minimum, the responsibility is on researchers and institutions to redesign processes that will invite and include non-researchers more fully in all aspects of making new knowledge.^11^

 A deeper consideration of equity in knowledge sharing would start with mutual acknowledgement that everyone has data collection skills and analysis skills—both university researchers and community members. Each brings knowledge about how to conduct a given project in a specific place. It does not mean that communities will or should acquire advanced statistical analytical skills, that communities’ ways of making knowledge become subject to validation by western science, or that researchers will or should fully understand multiple generations of knowledge from an indigenous community (for example). It means that spaces where research occurs see that various forms of knowledge are valuable and operationalizes this, perhaps purposefully compensating for the fact that knowledge from western research systems are generally considered more “legitimate” in research contexts.

 While the issue of valuing different types and sources of knowledge is well-argued in the literature, what is missing is an interrogation of what this looks like in practice. Pointedly, how will we know when we are valuing different ways of knowing and applying this knowledge meaningfully? Perhaps research projects begin to include outputs that are explicitly derived from different knowledge types and approaches – with community members supported and encouraged to develop their own findings from data in ways that resonate with them. The “co” part of coproduction in this sense, therefore, might be mutual learning and interrogation of findings by both community partners to researchers and vice versa. Communities have designed, implemented and published their own research in various topics and an equitable coproductive space might involve institutions acknowledging this research as relevant forms of knowledge—for example, by paying for community-led research reports as for academic journals.

*Redistributing decision-making:* Engaging in redistributive coproduction means re-thinking and re-allocating who holds decision-making power within a research program. Equal coproduction shares decision-making about key aspects of research between all parties. Attending to equity, however, recognizes inherent power differences that exist between researchers and non-researchers and reorganizes decision-making to attend to those differences. Equitable decision-making means, as a start, listening closely to marginalized communities and being willing to honor their requests, even if—especially if—those requests are uncomfortable. For example, a group might want to share power equally, but another might want to have their preferences weighted more heavily—let’s say through veto-power or overrepresentation—in decision-making processes. Committees composed solely of non-researchers where community members can debate more freely might be developed to solely govern certain aspects of the research including, for example, the research questions or deciding how grant monies will be allocated. While there is growing application of research projects including lived experience committees and other coproduction models, there needs to be more reporting and scrutiny of what roles these groups hold, how they operate, and how they are given and/or wield power in research endeavors.

## Future Considerations

 Thus far, we have spent most of our critique on institutional and project-level barriers to equitable coproduction. But equally important is the role of researchers working in an equitable, and disruptive, coproduction space. Many have discussed the importance of reflexivity as part of researchers’ practice^12^ and arguably, some of the most useful scholarship comes from reflexivity work. Author reflexivity and contribution statements in academic journals are becoming more common as understanding grows of the value these statements provide in aiding interpretation. Statements delineating author roles, identities and contributions are particularly important in coproduced research as a way to communicate the roles and contributions of non-researcher partners. Although these are steps forward, more can be done.

 In addition to reflecting back or currently on ourselves, our motivations, our decisions and actions, we need to start thinking forward. This requires, in part, imagining what future more fully realized coproduced research could look like and contending with the difficult questions these imagined futures will raise for those who currently hold power. For example, as researchers transfer knowledge and skills and learn from their community partners, how does their role change over time – especially when partners become equally skilled in research? Do researchers develop a new set of skills, maybe focusing on fundraising for projects, presenting findings in spaces where community members may not have access, or develop political advocacy expertise? Or maybe they are running errands, making sure audio/visual works, etc? What if the topic the researcher studies is no longer prioritized by the community with which they work? Should white people stop being researchers in or with Black, Indigenous and People of Color (BIPOC) communities as BIPOC communities build their own academic power and skills such that are recognized by research institutions? These are some of the questions that we have had to ponder in our own work.

 Imagining what a fully realized equitable coproduction model of research looks like requires researchers and research institutions, white ones in particular, to sit with deep uncertainty. Certainty plays a foundational role in maintaining concentrations of power, and equally communicates to people who are marginalized that nothing will change.^13^ For researchers and their institutions, to sit with uncertainty means a long-term commitment to engaging in imagining and enacting coproduction with partners where the future is unknown. This unknowingness is central to decolonization and anti-oppression work and requires us to explore collaboratively with others. It is an argument for committing to coproduction as a long-term way of working that extends far beyond the life of most research projects and their funds, but is not out of scope given the longevity of research institutions. If coproduction is to extend years, decades even, how might we reimagine how institutions support such a future—including how funding is allocated, who is considered a researcher, how researchers and community partners are trained, and how marginalized groups might become central power holders? It could mean, for example, prioritizing BIPOC for graduate student training spaces to become researchers with and in their own communities. In the United States, these types of prioritization conversations akin to affirmative action make people in white-dominant spaces uncomfortable. But there is precedence elsewhere for this kind of reimagining and redistribution.^14^

 It could also mean thinking differently about what it means to be in working relationships with community members who bring their lived expertise to coproduction research. Thus far, we have written from a perspective that accepts that research and its needs are important, but in imagining a fully equitable coproduction future, we must ask what are the community’s needs and how might researchers and their institutions address them? In coproduction, the sometimes hard realities of people’s lives not only blend with the research “work” space, but are often the very reason they are invited to or “qualified” for the particular work, eg, people experiencing homelessness, people living with a disability, etc. When partners leave the research space, they do not also leave the reality of their situations – as a researcher might. When this is the case, how should researchers think about their duties and moral obligations to their research partners, particularly when those partners are very vulnerable populations? Is it enough to provide financial compensation for work, knowing that such compensation may not be enough, desired, or even, an encumbrance rather than a support? The onus must not lie only within the purview of the lone researcher grappling with their moral compass; institutions must examine their roles, how their policies influence researchers’ actions, and their own moral obligations to communities with whom they seek to be in—and benefit from—research relationships.

 A long-term and uncertain commitment to coproduction does not mean that marginalized community members have a crystal-clear image of this coproduced research future. Not everyone in a community group wants to work on a research project, wants to learn a lot about the research process or how researchers operate. They may not even want a significant say over how a project is run. But, they (and we) want to see impact and a purpose for participating and therefore, these changes are well imagined. Imagining a more equitable model for coproduction research would create a research space where more power is held by historically marginalized groups, in all areas of the research. Played out to the end, a fully realized and equitable coproduction future might be a community space that produces research valued by academic and research institutions, and where researchers are invited in when appropriate. Something far less like collaborative research, and much more like self-determination.

## Conclusion

 The ideas we explore in this commentary are intended to provoke deeper thinking and creativity into how coproduction can be fully realized, but these ideas are not solely theoretical. There are examples of institutions, researchers and communities across the world currently challenging and successfully redistributing power in research environments. But academic publications about coproduction projects too often only allude to the problems that teams contended with in redistributing power. Perhaps there are more examples and learning that could be shared about the sticky, difficult questions coproduction teams have contended with and how they have been resolved—or perhaps as importantly, not resolved resulting in failed coproduction projects. Without open and robust discussion of such examples, the field may continue to (falsely) believe that coproduction is still a fledgling endeavor, resulting in more think pieces and calls to action, rather than a strategic approach to dismantling systemic barriers.

 We encourage more brave and bold discussion of the difficult issues teams grapple with in attending to equity, and how they are resolved to help others working in this space. We offer the idea of “futures thinking”—ie, imagining possible potential futures for coproduction and then strategically acting to bring about desired changes^15^—as a tool to identify specific approaches to change institutions in ways that will make coproduction more equitable and thus, more effective. This brings the discussion back to impact. If the goal of coproduction is to create new knowledge that drives change that has, thus far, eluded researchers doing research “as usual” – ie, working mostly within the confines of academic communities – then it must also be the goal of coproduction to change what is “usual” research – ie, by operationalizing research processes that are owned and driven by voices that have been systematically and intentionally excluded.

## Acknowledgements

 The authors thank Marisa Westbrook for feedback on an early draft of this commentary.

## Ethical issues

 Not applicable.

## Conflicts of interest

 Authors declares that they have no conflicts of interest.

## Endnotes


^[1]^ Written in 1969, the original quote used language for BIPOC identities that is now considered disrespectful or offensive. We have adapted the quote to retain the meaning without the harmful language. The full quote is easily discovered online for those who are interested.
^[2]^ In this commentary we consider patients as part of this group.
^[3]^ Notably, the authors did not have the ability to collaborate with a community member to write this paper—which would have been best practice—because we do not have funding to support a standing committee or non-project specific, ongoing role.
